# Effect of *Claroideoglomus etunicatum* and Indole-3-acetic Acid on Growth and Biochemical Properties of Vetiver Grass (*Vetiveria zizanioides*) Under Salinity Stress

**DOI:** 10.3390/ijms26073132

**Published:** 2025-03-28

**Authors:** Negar Mosallanejad, Mehdi Zarei, Reza Ghasemi-Fasaei, Amir Ghaffar Shahriari, Afsaneh Mohkami, Tibor Janda

**Affiliations:** 1 Department of Soil Science, School of Agriculture, Shiraz University, Shiraz 7144165186, Iran; negarmosallanejad93@gmail.com (N.M.); ghasemif@shirazu.ac.ir (R.G.-F.); 2 Department of Agriculture and Natural Resources, Higher Education Center of Eghlid, Eghlid 7381943885, Iran; shahriari.ag@eghlid.ac.ir; 3 Research and Technology Institute of Plant Production, Afzalipour Research Institute, Shahid Bahonar University of Kerman, Kerman 7616913439, Iran; amohkami@uk.ac.ir; 4 Centre for Agricultural Research, Agricultural Institute, Brunszvik u. 2., H-2462 Martonvásár, Hungary

**Keywords:** salinity, antioxidative enzymes, arbuscular mycorrhiza, vetiver

## Abstract

Salinity represents a major environmental factor limiting plant growth and productivity. In order to better understand the effects of arbuscular mycorrhizal fungus *Claroideoglomus etunicatum* and Indole-3-acetic acid (IAA) on the growth and chemical composition of vetiver grass (*Vetiveria zizanioides*) under salt stress, a factorial experiment was conducted in a completely randomized design with three replications. The experiment included four NaCl levels (0, 8, 16, and 24 decisiemens per meter (dS/m)) and four levels of treatments (no amendment application, application of IAA, application of *C. etunicatum*, and interaction of IAA and *C. etunicatum*) with three replications. The results of the experiment showed that the addition of sodium chloride increased the concentration of proline and the activities of catalase, peroxidase, and superoxide dismutase enzymes. The application of the growth regulator (IAA) and *C. etunicatum* significantly increased the fresh and dry weight (101%) of shoots, dry weight of roots, and the concentration of macro- and micro-elements in shoots under salinity condition (99.82% phosphorus; 9.79% Iron). The application of mycorrhiza and auxin significantly reduced the concentration of proline and the activities of catalase, peroxidase, and superoxide dismutase enzymes. In general, the addition of IAA and *C. etunicatum* to roots under salt stress conditions can improve growth and increase the concentration of some nutrients in vetiver shoots.

## 1. Introduction

More than 6% of the world’s land area and 20% of the world’s irrigated land are exposed to salinity [1]. Salinity represents a major abiotic stress severely affecting crop production, which is even more important in arid and semi-arid climates such as Iran [2]. In the search for a solution to combat salinity stress, the use of symbiotic microorganisms such as mycorrhizal fungi or plant growth-promoting bacteria has been proposed as a promising method [3,4]. Mycorrhiza represents a type of mutually beneficial symbiosis that occurs between plant-absorbing organs (usually roots) and hyphae of certain fungi. In general, both symbiotic components benefit from this symbiosis [5]. Mycorrhiza plays a key role in the rhizosphere, as they act as an important link in the exchange of nutrients between plants and rhizosphere soil. In this process, mycorrhizal fungi improve both plant nutrition and soil stability. The fungi enhance plant growth by absorbing minerals from the soil and making plants more resistant to stress [6]. Mycorrhizal fungi play a vital role in enhancing plant growth by forming a symbiotic relationship with plant roots, often referred to as mycorrhizae [7]. These fungi extend their hyphae into the soil, increasing the surface area for nutrient absorption far beyond what plant roots can achieve alone. Through this network, mycorrhizal fungi efficiently absorb essential soil minerals such as phosphorus, nitrogen, and micronutrients, which might otherwise be unavailable to plants due to their low mobility in soil. The fungi regulate nutrient uptake by the host plant in exchange for carbohydrates produced during photosynthesis, fostering mutualistic benefits [8].

The result of this function is better plant growth and, consequently, an increase in carbon sources in the soil, which increases the activity of soil microorganisms; microbial metabolites improve soil structure and increase the plant’s ability to resist/tolerate stress [9]. Under saline conditions, the arbuscular mycorrhizal fungi trigger various mechanisms enabling the crop to cope with salinity stress [10]. These include saving stability of cellular membranes [11], more efficient minerals uptake [12], improving photosynthesis [13], and water use efficiencies [14]; all of which are crucial for crop survival under salinity stress. Among the diverse types of mycorrhizal fungi, *Claroideoglomus etunicatum* stands out for its unique contributions to plant growth and soil health. This arbuscular mycorrhizal fungus significantly enhances plant tolerance to environmental stresses such as salinity and boron toxicity [13]. By forming a symbiotic relationship with plant roots, this fungus improves nutrient uptake, particularly phosphorus and potassium, while reducing the absorption of harmful ions like sodium and boron under stress conditions. This nutrient optimization strengthens the plant’s physiological processes, such as chlorophyll production and photosynthesis, which are crucial for growth and stress resilience [14]. Additionally, *C. etunicatum* boosts the plant’s antioxidant defense system by increasing the activity of enzymes like superoxide dismutase and catalase. These enzymes mitigate oxidative damage caused by stress, ensuring cellular stability [15]. The fungus also enhances water retention and osmotic regulation in plant tissues, further supporting growth under adverse conditions. Through these mechanisms, *C. etunicatum* plays a vital role in maintaining plant health and productivity in challenging environments [16].

In addition to symbiotic microorganisms, plant growth regulators are also involved in increasing plant resistance to abiotic stresses including salinity [17]. Auxin (IAA, indole-3-acetic acid) is one of the most important plant hormones that regulate plant growth and development. Various studies have shown that exogenous application of IAA improves plant resistance under salinity stress [18,19]. For example, it has been shown that exogenous application of IAA drives a wide range of physiological and developmental processes, including stomatal opening, aquaporin production, and accumulation of osmotic regulators, such as proline and soluble sugars, and thus increases plant resistance to stress [20]. In addition, auxin helps plants to tolerate stress by scavenging reactive oxygen species, reducing Na^+^ accumulation, and protecting photosystem II (a protein–pigment complex in the thylakoid membranes of chloroplasts that captures light energy to drive the initial stages of photosynthesis) from damage [21]. It has also been reported that the exogenous application of IAA stimulates root system development, ultimately leading to increased resistance to salinity stress [22]. Vetiver grass (*Vetiveria zizanioides*), belonging to the Poaceae family, is a perennial plant used to improve degraded land and also as fodder for livestock [23]. The plant can adapt to many regions of Iran where it can play a great role in watershed management to reduce soil erosion [24]. Also, there have been studies reporting improved growth of vetiver as inoculated by microorganisms [18,25]. Vetiver grass plays a crucial role in sustainable land management due to its deep root system, which prevents soil erosion and stabilizes slopes. It is highly effective in water conservation and remediation of contaminated lands, making it valuable for environmental restoration. Additionally, its versatility extends to agriculture, bioengineering, and even aromatic oil production, offering diverse economic and ecological benefits [26].

Although halophytes are more resistant to salt stress than other plants, their growth and performance are also negatively affected by salinity [27]. Therefore, in recent years, efforts have been made to increase the resistance of halophytes to salt stress [28]. On the other hand, such studies provide improved insight into the mechanisms by which microorganisms contribute to increased tolerance to salt stress. While studies have shown positive effects on root system development [29], levels of other hormones such as gibberellins [26], and improved nutrient uptake [30], it is not clear whether auxin directly triggers the plant’s defense system against salt stress. Therefore, more research is needed to better understand the role of auxin in increasing plant resistance to salt. Also, given that auxin is involved in various intracellular mechanisms [31], thus, the present study was carried out to investigate the effect of the arbuscular fungus *C. etunicatum* and Indole-3-acetic Acid on growth and biochemical properties of vetiver grass under salinity stress.

## 2. Results

### 2.1. Effect of NaCl, IAA and C. etunicatum on Morphological Properties and Biomass Production of Vetiver

**Figure 1:**
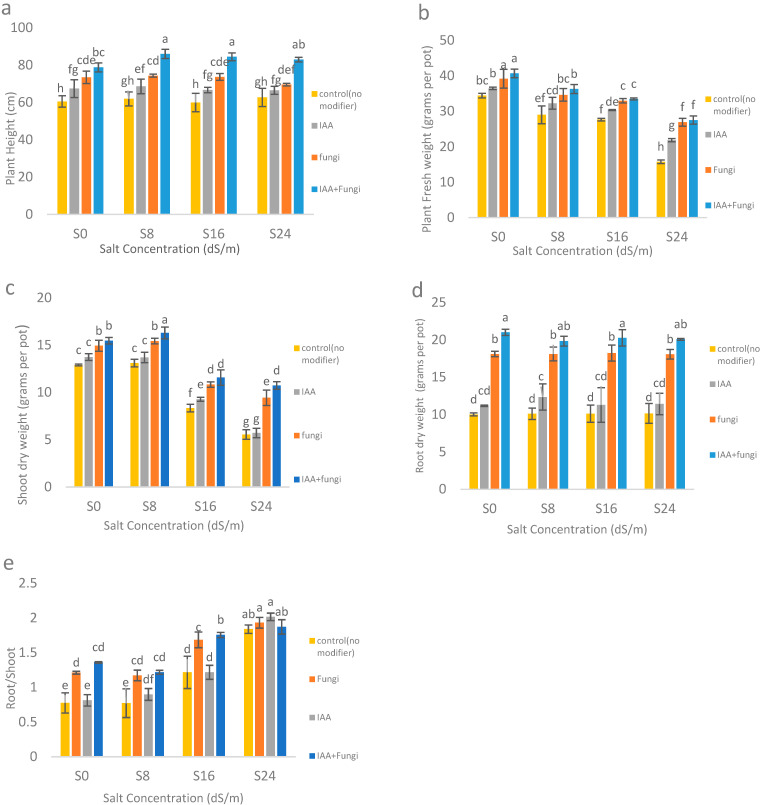
Effect of salinity levels, IAA, and the *Claroideoglomus etunicatum* on growth characteristics and yield of vetiver. (**a**) Plant growth as affected by salinity levels, IAA, and *C. etunicatum* inoculation (data represented are average from all replications); (**b**) Impact of salinity levels, IAA, and *C. etunicatum* on shoot fresh weight measured as grams per pot; (**c**) Impact of salinity levels, IAA, and *C. etunicatum* inoculation on shoot dry weight measured as grams per pot; (**d**) Impact of salinity levels, IAA, and *C. etunicatum* on root dry weight measured as grams per pot; (**e**) Root to shoot ratio. S0, S8, S16, and S24 mean 0, 8, 16, and 24 dS/m of NaCl. The letters shown on the top of bars represent statistically significant differences.

The application of NaCl increased the plant height, but this increase was not significant. The application of modifying treatments (IAA and *C. etunicatum*) significantly increased the plant height. The average plant height increased with the application of IAA, fungus, and the combined application of IAA and fungus compared to the control treatment (Figure 1a).

The effect of NaCl, the modifying treatments, and their interaction on shoot fresh weight was significant (*p* < 0.01) (Table A1). With the increase in NaCl, a significant difference in shoot fresh weight was observed (Figure 1b). The application of NaCl at 8, 16, and 24 dS/m significantly reduced the average shoot fresh weight compared to the control by 12.34, 17.44, and 38.97%, respectively. The application of IAA and *C. etunicatum* also increased the shoot fresh weight. The application of IAA, fungus, and the interaction of IAA and fungus significantly increased the average shoot fresh weight compared to the control by 13.18, 25.14, and 29.14%, respectively, while no significant difference was observed between the levels of *C. etunicatum* and IAA+*C. etunicatum* interaction. The highest fresh weight of vetiver shoots was observed in “no salinity” and the interaction of IAA+*C. etunicatum*. The lowest shoot fresh weight was related to 24 dS/m NaCl without the application of corrective treatments.

Besides the effect of NaCl, IAA, *C. etunicatum*, and their interaction on the dry weight of the plant shoot was significant (Figure 1c). While the application of 8 dS/m did not cause substantial change, with the application of NaCl 16 and 24 dS/m, the average shoot dry weight decreased by 29.82 and 44.91% compared to the control treatment, respectively. The application of IAA and *C. etunicatum* treatments also increased the shoot dry weight. The application of IAA, *C. etunicatum*, and their interaction significantly increased the average shoot dry weight by 6.37, 26.94, and 35.57% compared to the control treatment, respectively (as an average of all the treatments). The highest shoot dry weight was related to NaCl 8 dS/m and the combined application of IAA and *C. etunicatum*. Also, the lowest shoot dry weight was observed in NaCl 24 dS/m without the application of the IAA and fugal treatments.

The results showed that the impact of modifying treatments (IAA and *C. etunicatum*) was significant on the dry weight of vetiver plant roots (*p* < 0.01). According to mean comparison, the application of IAA and *C. etunicatum* treatments also increased the dry weight of the plant roots. The application of IAA, *C. etunicatum*, and the interaction of IAA and *C. etunicatum* increased the average dry weight of the plant roots by 14.48, 79.76, and 101%, respectively, compared to the control.

### 2.2. Effect of NaCl, IAA, and C. etunicatum on the Nutritional Elements of Vetiver

The effects of salinity, IAA, and *C. etunicatum* and their interaction on shoot sodium concentration were significant. With the application of salinity of 8, 16, and 24 dS/m, the average shoot sodium concentration was 1.17, 2.13, and 4.15 times that of the control treatment, respectively. With the application of salinity of 8, 16, and 24 dS/m, the average total shoot sodium uptake was 1.20, 1.50, and 2.40 times that of the control treatment, respectively. The application of IAA and *C. etunicatum* treatments also increased the total shoot sodium uptake. Thus, the application of *C. etunicatum* and the interaction of IAA and *C. etunicatum* significantly increased the average total shoot sodium uptake by 38.25 and 50.06%, respectively, compared to the control treatment. The highest total shoot sodium uptake was related to salinity of 24 dS/m, interaction of IAA, and *C. etunicatum* amendment treatments. Also, the lowest total shoot sodium uptake was related to no application of sodium chloride and no application of growth regulators. The effect of NaCl and the interaction effect of salinity levels and amendment treatments at the 1% level on total root sodium absorption was significant. With the application of salinity of 8, 16, and 24 dS/m, the average total root sodium absorption was 5.62, 10.76, and 17.43 times that of the control treatment, respectively. The application of IAA and *C. etunicatum* treatments also caused a decrease in total root sodium absorption. However, this decrease was not significant. The highest total root sodium absorption was related to the treatment of salinity level of 16 dS/m and the interaction of growth regulator and *C. etunicatum*. The lowest total root sodium absorption was related to the surface without salinity stress and without the application of amendment treatments (growth regulator and *C. etunicatum*) (Table 1).

The average shoot potassium concentration at different levels of sodium chloride, amendment treatments, and their interaction was significant (*p* < 0.01). The application of sodium chloride caused a significant decrease in shoot potassium concentration and the average shoot potassium concentration at salinities of 8, 16, and 24 dS/m decreased by 0.38, 14.66, and 21.60% compared to the control treatment, respectively. The application of IAA and *C. etunicatum* treatments also caused a significant increase in shoot potassium concentration. So, the average shoot potassium concentration with the application of *C. etunicatum* and the interaction of IAA and *C. etunicatum* increased by 0.14 and 0.57% compared to the control treatment, respectively.

With increasing salinity, a significant difference was observed in the average total shoot potassium absorption. The application of 16 and 24 dS/m salinity caused a 40.14 and 56.81% decrease in total shoot potassium absorption compared to the control treatment, respectively. The application of IAA and *C. etunicatum* treatments also caused an increase in the average total shoot potassium absorption. Thus, the application of IAA, *C. etunicatum*, and the interaction of IAA and *C. etunicatum* significantly increased the average total shoot potassium absorption compared to the control treatment by 18.6, 25.64, and 34.32%, respectively. The application of sodium chloride significantly reduced the root potassium concentration, and the average root potassium concentration decreased by 10.90, 17.86, and 32.94% in salinities of 8, 16, and 24 dS/m, respectively, compared to the control treatment. The application of IAA and *C. etunicatum* treatments also significantly increased the root potassium concentration. The average root potassium concentration increased by 1.11, 2.50, and 2.78% with the application of IAA, fungus, and the interaction of IAA and *C. etunicatum*, respectively, compared to the control treatment. However, no significant difference was observed between the application levels of *C. etunicatum*, growth regulator interaction, and *C. etunicatum*. The highest root potassium concentration was related to the level without salinity stress and the interaction of IAA and *C. etunicatum* (Table 2).

The effects of salinity, IAA, and mycorrhiza and their interaction on the average phosphorus concentration of the plant’s aerial parts were significant (*p* < 0.01). At salinities of 8, 16, and 24 dS/m, the average phosphorus concentration of the aerial parts decreased by 6.58, 19.16, and 38.32%, respectively, compared to the control treatment. The application of IAA and *C. etunicatum* treatments increased the average phosphorus concentration of the aerial parts; so the application of IAA, *C. etunicatum*, and the interaction of IAA and *C. etunicatum* significantly increased the average phosphorus concentration of the aerial parts compared to the control treatment by 0.71, 1.43, and 2.15%, respectively. The highest phosphorus concentration of the aerial parts was related to the unstressed surface and the interaction of IAA and *C. etunicatum*. The lowest phosphorus concentration of the aerial parts was related to the salinity of 24 dS/m and without IAA and mycorrhiza treatments.

The effect of salinity, IAA, and mycorrhiza on the average total phosphorus uptake of the plant’s aerial parts was significant (*p* < 0.01), while their interaction was not significant. The application of salinity of 8, 16, and 24 dS/m caused a decrease of 4.06, 43.17, and 66.95% of the total phosphorus uptake of the aerial parts compared to the control treatment, respectively. The application of IAA and *C. etunicatum* treatments also caused an increase in the average total phosphorus uptake of the aerial parts. Thus, the application of IAA, *C. etunicatum*, and the interaction of IAA and *C. etunicatum* significantly increased the average total phosphorus uptake of the aerial parts compared to the control treatment by 7.32, 26.42, and 35.04%, respectively (Table 3).

The effect of salinity, IAA, and *C. etunicatum* on the iron concentration of the shoot was significant. The application of sodium chloride significantly reduced the iron concentration in the shoot, and the average iron concentration of vetiver shoots at salinities of 8, 16, and 24 dS/m was reduced by 18.86, 30.82, and 47.31% compared to the control treatment, respectively. The application of IAA, *C. etunicatum*, and their interaction increased the average iron concentration of the shoot by 19.5, 99.1, and 81.6% compared to the control treatment, respectively. The highest iron concentration in the shoot was related to the surface without salinity stress and the interaction of IAA and *C. etunicatum*. Also, the lowest iron concentration in the shoot was related to the surface of 24 dS/m and without the application of the amendment.

The effect of NaCl and amendment treatments and their interaction on the average zinc concentration in the plant shoots was with the application of salinity of 8, 16, and 24 dS/m, and the average concentration of zinc in the shoot increased by 5.28, 61.42, and 98.84% compared to the control treatment, respectively. The application of IAA and *C. etunicatum* treatments also increased the average concentration of zinc in the shoot. Thus, the application of IAA, *C. etunicatum*, and the interaction of IAA and *C. etunicatum* significantly increased the average concentration of zinc in the shoot compared to the control treatment by 5.05, 1.90, and 7.09%, respectively.

The effect of salinity, IAA, and *C. etunicatum* and their interaction on the concentration of Zn in the root was significant. The application of sodium chloride significantly reduced the root zinc concentration, and the average root zinc concentration decreased by 21.02, 43.14, and 64.51% in salinities of 8, 16 and 24 dS/m, respectively, compared to the control treatment. The application of IAA and *C. etunicatum* treatments also significantly increased the root zinc concentration. The average root zinc concentration increased by 2.65, 1.62, and 4.33% in the case of IAA, *C. etunicatum*, and the interaction of IAA and *C. etunicatum*, respectively, compared to the control treatment.

The effect of NaCl and the amendment treatments on the average copper concentration of the plant’s aerial parts was significant, while their interaction was not significant. With the application of salinity of 8, 16, and 24 dS/m, the average copper concentration of the aerial parts decreased by 17.02, 30.68, and 49.67% compared to the control treatment, respectively. The application of IAA and *C. etunicatum* treatments also increased the average copper concentration of the aerial parts. Thus, the application of IAA, *C. etunicatum*, and the interaction of IAA and *C. etunicatum* significantly increased the average copper concentration of the aerial parts compared to the control treatment by 3, 1.85, and 5.37%, respectively.

The effect of salinity, IAA, and *C. etunicatum* and their interaction on the copper concentration of vetiver roots was significant. The application of salinity of 8, 16, and 24 dS/m caused a significant decrease in root copper concentration by 24.41, 42.40, and 59.79%, respectively, compared to the control treatment. IAA and *C. etunicatum* treatments also caused a significant increase in root copper concentration. The average root copper concentration increased by 3.13, 1.83, and 3.86%, respectively, compared to the control treatment with the application of IAA, *C. etunicatum*, and the interaction of IAA and *C. etunicatum*. However, no statistically significant difference was observed between the levels of growth regulators and mycorrhiza and between the application of IAA and *C. etunicatum*.

The average concentration of shoot manganese was significant at different levels of sodium chloride, amendment treatments, and their interaction. The application of sodium chloride significantly reduced the concentration of manganese in the shoot, and the average concentration of manganese in the shoot decreased by 4.04, 29.36, and 47.08% compared to the control treatment at salinities of 8, 16, and 24 dS/m, respectively. The application of IAA and *C. etunicatum* treatments also significantly increased the concentration of manganese in the shoot. The average concentration of manganese in the shoot increased by 2.37, 7.33, and 11.24% compared to the control treatment with the application of IAA, *C. etunicatum*, and the interaction of IAA and *C. etunicatum*, respectively (Table 4).

The effect of salinity, IAA, and *C. etunicatum* and their interaction was significant at the 1% level on the concentration of manganese in vetiver roots. The application of salinity of 8, 16, and 24 dS/m caused a significant decrease in the root manganese concentration by 18.08, 36.53. and 58.23%, respectively, compared to the control treatment. The application of IAA and *C. etunicatum* treatments also caused a significant increase in the root manganese concentration. The highest root manganese concentration was related to the surface without salinity stress and IAA–mycorrhiza interaction. The lowest root manganese concentration was related to the salinity of 24 dS/m and without the application of the amendment treatment (Table 5).

### 2.3. Effect of NaCl, IAA, and C. etunicatum on the Antioxidative Responses of Vetiver

The effect of salinity levels, IAA, and *C. etunicatum* and their interaction on plant proline concentration is significant. The application of salinity of 16 and 24 dS/m caused a significant increase in plant proline concentration by 62.58 and 86.88%, respectively, compared to the control treatment. The application of IAA, *C. etunicatum*, and their interaction treatments caused a decrease in plant proline concentration. The average plant proline concentration decreased by 6.40, 11.71, and 14.30%, respectively, compared to the control treatment with the application of IAA, *C. etunicatum*, and the auxin–mycorrhiza interaction. The highest plant proline concentration was related to the salinity level of 24 dS/m and the absence of the application of the corrective treatments. The lowest plant proline concentration was related to the salinity of 8 dS/m and the interaction of IAA and *C. etunicatum*.

The effect of NaCl, IAA, and *C. etunicatum* and their interaction on plant catalase enzyme activity is significant. The application of salinity of 8, 16, and 24 dS/m caused a significant increase in plant catalase enzyme activity by 14.32, 20, and 38.45%, respectively, compared to the control treatment. The application of IAA, *C. etunicatum*, and their interaction caused a decrease in plant catalase enzyme activity. So the average plant catalase enzyme activity decreased by 5.32, 13.18, and 18.78%, respectively, compared to the control treatment with the application of IAA, *C. etunicatum*, and the interaction. The highest level of plant catalase enzyme activity was related to the salinity level of 24 dS/m and without the application of amendment treatments. The effect of NaCl (salinity) on superoxide dismutase enzyme activity is significant. The application of NaCl increased the activity of superoxide dismutase enzyme in vetiver. The application of salinity of 16 and 24 dS/m significantly increased the activity of superoxide dismutase enzyme in vetiver, by 44.32 and 69.56%, respectively, compared to the control treatment. The application of modifying treatments decreased the activity of the superoxide dismutase enzyme, but this decrease was not significant.

The effect of salinity levels, IAA, and *C. etunicatum* and their interaction on the activity of peroxidase enzyme in vetiver is significant. The application of NaCl increased the activity of superoxide dismutase enzyme in vetiver. The application of salinity of 8, 16, and 24 dS/m significantly increased the activity of peroxidase enzyme in vetiver by 13.62, 30.18, and 42.41%, respectively, compared to the control treatment. The use of IAA, *C. etunicatum*, and the interaction of these two treatments also caused a decrease in the activity of peroxidase enzyme. The average activity of peroxidase decreased by 4.75, 5.27, and 9.40% with the use of IAA, *C. etunicatum*, and the interaction of IAA and *C. etunicatum*, respectively, compared to the control. The highest level of plant peroxidase enzyme activity was related to the salinity level of 24 dS/m and without the use of corrective treatments. The lowest level of plant peroxidase enzyme activity was related to the level without salinity stress and the interaction of IAA and *C. etunicatum* (Table 6).

## 3. Discussion

The results showed that *C. etunicatum* treatment significantly increased plant height, fresh weight, and dry weight. The mycorrhizal fungus enhances plant height, fresh weight, and dry weight by improving nutrient uptake and overall plant health. This fungus forms a symbiotic relationship with plant roots, extending its hyphal network into the soil to absorb essential nutrients like phosphorus, nitrogen, and potassium. These nutrients are then delivered to the plant, promoting robust growth and increasing plant height. This is in line with the results reported previously [15]. Moreover, *C. etunicatum* contributes to the accumulation of biomass, reflected in increased fresh and dry weight. By improving root architecture and nutrient efficiency, the fungus ensures that plants have access to the resources needed for sustained growth. Moreover, as mentioned elsewhere, enhanced photosynthetic activity and reduced stress levels, facilitated by the fungus, also play a role in boosting biomass production [8].

Arbuscular mycorrhizal symbiosis has been shown to improve nutrient uptake by up to 80% which lowers the detrimental impact of salinity stress [32]. So the improved growth can be attributed to increased absorption of nutritional elements under salt stress conditions because mycorrhiza fungi significantly increase the availability of nutrients and thus reduce the detrimental effects of sodium on plant growth [33]. Exogenous application of IAA drives a wide range of physiological and developmental processes, including stomatal opening, aquaporin production, and accumulation of osmotic regulators, such as proline and soluble sugars, and thus increases plant resistance to stress [20]. Based on the results, the application of mycorrhiza and/or IAA applications significantly increased the fresh and dry weight of the plant.

Mycorrhizal symbiosis may also enhance the content of organic acids in plants which lowers soil electrical conductivity and increases the availability of nutritional elements in soil [9]. Moreover, it has been shown that mycorrhizal symbiosis promotes polyamine contents in plants that maintain homeostasis in plant cells by enhancing the uptake of nutrients and water [34]. In accordance with these, it was also found that *C. etunicatum*, IAA, and their interaction treatments had a positive and significant effect on improving nutrient absorption. Regarding the complementary role of IAA, it should be noted that exogenous application of IAA (here IAA) improves the growth and development of adventitious root by which uptake of nutritional minerals can be accelerated. Previous studies also highlight the role of IAA and other IAA types in the improvement of adventitious root and increased mineral uptake [35]. As a whole, better growth and micronutrient uptake by arbuscular mycorrhizal symbiosis and IAA application may be attributed to a widespread root–hyphal system that shortens the path of nutrients’ uptake, better binding of nutrients to fungal mycelium, and also to changes in soil pH which improves nutrient solubility and availability [22]. Overall, the application of IAA and *C. etunicatum* not only reduces proline accumulation by enhancing stress tolerance mechanisms but also modulates the activities of key antioxidant enzymes. This dual action helps mitigate the detrimental effects of reactive oxygen species (ROS) under high salinity conditions, showcasing a comprehensive approach to improving plant resilience.

IAA enhances the physiological resilience of vetiver grass to salt stress by promoting root elongation and increasing root surface area, which improves water and nutrient uptake under saline conditions. This hormone also regulates stomatal behavior, reducing water loss and maintaining cellular turgor [31]. Biochemically, IAA stimulates the production of osmoprotectants like proline and glycine betaine, which help in osmotic adjustment. It also enhances the activity of antioxidant enzymes such as catalase and superoxide dismutase, mitigating oxidative damage caused by reactive oxygen species (ROS) under salt stress [36]. *C. etunicatum*, the mycorrhizal fungus, complements these effects by forming symbiotic associations with vetiver roots, improving ion homeostasis and reducing sodium toxicity. At the molecular level, this fungus activates stress-responsive genes involved in ion transport and ROS detoxification [8]. It also enhances the synthesis of secondary metabolites and phytohormones, further boosting the plant’s defense mechanisms. Together, IAA and Claroideoglomus etunicatum create a synergistic effect, enabling vetiver grass to thrive in saline environments by optimizing physiological, biochemical, and molecular pathways [15].

Based on the results, in high salinity, the application of *C. etunicatum*, IAA, and their interaction reduced the proline content of the plant. IAA can decrease proline content by enhancing the plant’s stress tolerance mechanisms, reducing the need for proline accumulation [18]. For example, it has been reported that IAA reduced the proline content in cotton under salt stress [37]. It may also regulate the enzymes involved in proline metabolism, such as proline dehydrogenase (which degrades proline) and pyrroline-5-carboxylate synthetase, a key enzyme in the synthesis of proline.

In more detail, the synergy between the fungus *C. etunicatum* and IAA in mitigating the adverse effects of salt stress on vetiver is significant through their enhancement of plant physiological and molecular functions. This fungus improves nutrient absorption and regulates ion balance, preventing the accumulation of toxic ions such as sodium, while auxin promotes root growth, increasing the plant’s contact surface with the soil. Additionally, both factors stimulate the production of antioxidants and osmoprotectant compounds, reducing oxidative stress caused by salinity. This collaboration of these two biological stimulants strengthens plant salt tolerance and boosts the efficiency of the plant’s natural defense systems.

Reactive oxygen species (ROS) production is increased when plants are subjected to biotic or abiotic stresses. Both enzymatic and non-enzymatic mechanisms are involved in the alleviation of ROS detrimental effects [38]. Similarly to proline, high salinity induced the activities of certain antioxidant enzymes. However, the application of IAA, *C. etunicatum*, and their interaction caused a decrease in antioxidative enzymes including CAT, SOD, and peroxidase. Catalase represents a major component of an enzymatic ROS-mitigating mechanism whose activity in many plants under salinity is well documented [39,40]. However, CAT activity is not essentially increased in response to salinity and the enzyme activity depends on the dynamics of plant–stress interaction. For example, it has been reported that CAT activity is initially decreased at lower levels of salinity in *Cymbopogon nardus*, *Cynodon dactylon*, *Pennisetum alopecuroides*, and *Vetiveria zizanioide* [41]. In accordance with the present results, a decrease in catalase enzyme activity in barley seedlings inoculated with Azospirillium bacteria has also been reported [42]. It seems that mycorrhiza and IAA treatment reduced ROS production by improving nutritional conditions in the plant, thus reducing CAT activity. Also, mycorrhiza and IAA treatment may have a greater effect on the non-enzymatic mechanism of free radical removal.

It seems that under mycorrhizal treatment, the production of ROS decreased and, as a result, the activity of antioxidant enzymes decreased. In a study, inoculation of soybean plants with PGPR bacteria under salt stress conditions caused a significant decrease in the activity of antioxidant enzymes compared to control plants [43]. Furthermore, the presence of IAA either as an exogenous agent or produced by the microorganism may mitigate proline content or antioxidant enzyme activity. Superoxide dismutase converts superoxide into H_2_O_2_ and atomic oxygen and catalase and peroxidase enzymes convert H_2_O_2_, which is toxic to cells, into water and oxygen [44]. Therefore, under salinity stress, superoxide dismutase alone is not able to protect the plant against oxidative stress, and the increase in enzymes involved (catalase and peroxidases) in the detoxification of H_2_O_2_ is necessary [45]. A previous study demonstrated that IAA-producing bacteria could reduce the accumulation of proline and antioxidant enzymes such as superoxide dismutase (SOD) in wheat under salinity stress. This reduction in proline and antioxidant enzyme activity is attributed to the modulation of stress-responsive genes, which affected the plant’s overall stress tolerance and growth regulation [46]. Moreover, it has been found that IAA can lead to a reduction in the activity of antioxidant enzymes such as catalase (CAT) and ascorbate peroxidase (APX) under salinity stress. This reduction is attributed to the modulation of hormonal pathways that prioritize growth over stress defense [47].

## 4. Materials and Methods

### 4.1. Soil Collection and Experimental Setup

The soil was collected from a depth of 0 to 30 cm of limestone soil located in the Fars Bajgah area (longitude 52.5885706 and latitude 29.7199949 and altitude 1784 m). The samples were mixed with sand after air-drying and passing through a two-millimeter sieve. Physicochemical properties of the soil are presented in Table 7. The experiment was conducted in a greenhouse located in Bajgah, Fars Province, Iran. The temperature ranged between 20 and 35 °C, with a relative humidity of approximately 50–60%. The plants received full sunlight for 12–14 h per day. Irrigation was applied every 2 to 3 days, maintaining soil moisture up to field capacity.

### 4.2. C. etunicatum Inoculum Preparation

The initial inoculum of *C. etunicatum* was prepared in the Soil Science Department and applied at a rate of 60 g under a soil layer in pots designated for fungal treatment. To maintain uniform experimental conditions, an equivalent amount (60 g) of the substrate from fungus-free pots was added to the soil in the control treatments. The fungus *C. etunicatum* was inoculated in a layer of soil at a rate of 60 g in pots containing the fungus. In the control treatments without the fungus, 60 g of the substrate from the pots without the fungus was added to the soil to equalize the conditions for all pots.

Two experiments were conducted as a 3 × 4 × 4 factorial in a completely randomized design with three replications. The experiment included four levels of NaCl (0, 8, 16, and 24 dS/m) and four levels of amendment treatments [control, application of IAA, application of *C. etunicatum*, and combined application of IAA and the fungus]. Four kilograms of soil samples were prepared and placed in plastic bags after passing through a 4 mm sieve. The plants were obtained from the Ramhormoz region in Khuzestan Province, and 4 vetiver plants were planted in each pot. During the growth period, the plants were irrigated with distilled water at the field capacity. Four weeks after planting, salinity treatments were applied four times over four weeks. A week later, two liters of a 1 mM IAA solution were prepared and sprayed twice within an interval of one month. After 25 weeks, the plants were harvested and weighed. After weighing and washing, the plant samples were dried in an oven at 65 °C for 48. The dried samples were then weighed and powdered using an electric grinder for laboratory analysis. To measure plant enzymes, two grams of shoot fresh weight was taken, placed in a container containing liquid nitrogen, and stored in a −80 freezer.

To prepare the Indole-3-acetic acid (IAA) spray, 159 mg of IAA was accurately weighed and dissolved in 2.5 mL of ethanol. The solution was then diluted with distilled water to a final volume of 1 L to achieve the desired concentration. To prevent unintended exposure of the soil to the hormone, the surface was covered with black nylon bags, ensuring that the spray only reached the plants. The spraying process was conducted after sunset using a mist sprayer; 24 pots were treated with the IAA solution, while an additional 24 pots received distilled water as a control. This procedure was repeated after four weeks to maintain consistency in application. The spraying was specifically carried out at sunset to minimize the degradation of the hormone due to light exposure.

### 4.3. Nutrient Uptake Measurement

Soil mineral nutrients were measured by oxidation method with chromic acid, electrical conductivity in the saturated extract by electrical conductivity meter, usable phosphorus by extracting with sodium bicarbonate and measuring with spectrophotometer, concentration of cationic low-consumption elements (manganese, copper, zinc and iron) was determined by reading with an atomic absorption device (Shimadzu-AA670), and potassium was determined by extracting with ammonium acetate and reading with a flame photometer. Microbial respiration was measured using closed jars and carbon titration of microbial biomass was measured using the incubation–fumigation method.

### 4.4. Measurement of Proline and Antioxidant Enzyme Activities

Proline content was measured by the method proposed by Bates et al. (1973) [48], and the method developed by Ozden et al. (2009) was used to measure the activity of antioxidant enzymes [49].

#### 4.4.1. Measurement of Proline

The proline concentration was measured based on Bates et al. (1973) [48], starting with grinding 0.1 g of fresh plant powder in 10 mL of 3% sulfosalicylic acid to create a homogeneous mixture, which was then filtered. A 2 mL portion of the filtrate was mixed with 2 mL of ninhydrin reagent and 2 mL of glacial acetic acid, incubated at 100 °C for one hour, and cooled in an ice bath. After adding 4 mL of toluene and mixing vigorously, the samples were left to separate into phases, with the upper phase analyzed at 520 nm using a spectrophotometer; proline concentration (mg/g fresh weight) was determined via a standard calibration curve.

#### 4.4.2. Assessment of Antioxidant Enzyme Activity (Superoxide Dismutase (SOD), Catalase (CAT), and Peroxidase (POD)) in Plants

To prepare the extract required for assessing antioxidant enzyme activity, 0.5 g of fresh leaf tissue was homogenized in 2 mL of chilled 50 mM potassium phosphate buffer (pH = 7), containing 2 mM ethylenediaminetetraacetic acid (EDTA) and 1% (*w*/*v*) polyvinylpyrrolidone (PVP), collectively referred to as the extraction buffer.

The resulting homogenate was centrifuged at 10,000 rpm for 10 min at 4 °C using a refrigerated centrifuge (SIGMA 3-16PK, manufactured by SIGMA, Germany). The obtained supernatant was collected as the enzyme extract and stored at −80 °C until further enzymatic assays were performed [49].

##### Measurement of Superoxide Dismutase (SOD) Activity

The activity of superoxide dismutase (SOD) was measured using Ozden et al. (2009) [49], where a 3 mL reaction mixture was prepared containing enzyme extract, potassium phosphate buffer (pH 7.8), L-methionine, nitroblue tetrazolium chloride (NBT), EDTA, and riboflavin, which was freshly prepared in the dark. After mixing, the reaction mixtures were illuminated for 15 min with fluorescent lamps, followed by termination in darkness. A blank sample without enzyme extract served as the zero-percent reference, while a control sample exposed to light produced maximum color. Absorbance was recorded at 560 nm using a spectrophotometer, with enzyme activity expressed in units per milligram of fresh weight based on the Giannopolitis and Ries (1977) equation [50]:

$$\mathrm{SOD}\;\mathrm{Enzyme}\;\mathrm{Activity}\;(\mathrm{Unit}/\mathrm{mgFW})\;=\;\frac{\left[\frac{\left(\mathrm{OD}\;\mathrm{Control}\;-\;\mathrm{OD}\;\mathrm{Sampel}\right)\;\times \;100}{50}\right]}{\mathrm{Dilition}\;\mathrm{factor}\;\times \;\mathrm{FW}}$$

where:

OD represents the absorbance of the control and sample,

FW is the fresh weight of the plant sample, and

The dilution factor accounts for any sample dilution.

##### Measurement of Catalase (CAT) Activity

Catalase (CAT) activity was assessed based on the decrease in absorbance due to the decomposition of hydrogen peroxide at a wavelength of 240 nm, using a spectrophotometer (JENWAY 7315, UK). The measurements were taken over a 1 min period at 10 s intervals [49]. The reaction mixture (3 mL) contained 50 µL of the extracted enzyme solution, 50 mM potassium phosphate buffer (pH = 7), and 10 mM hydrogen peroxide. The enzyme activity was calculated using the extinction coefficient (Ɛ = 34.9 mM^−1^cm^−1^) and expressed as micromoles of hydrogen peroxide decomposed per minute per gram of fresh weight (FW), using the following formula:

$$\mathrm{CAT}\;\mathrm{Enzyme}\;\mathrm{Activity}\;({\mathrm{Mm}}^{-1}{\mathrm{cm}}^{-1}\mathrm{FW.min})=\frac{\mathrm{OD}(n)\;-\;\mathrm{OD}(0)}{\mathrm{Dilition}\;\mathrm{factor}\;\times \;\mathrm{FW}\;\times \;\mathrm{time}\;\times \;Ɛ}$$

where:

OD(n) and OD(0) represent the absorbance at time n and time zero, respectively,

FW is the fresh weight of the sample,

The dilution factor accounts for any sample dilution,

Time represents the reaction duration in minutes, and

Ɛ is the extinction coefficient of hydrogen peroxide at 240 nm.

##### Measurement of Peroxidase Enzyme Activity (POD)

The activity of the peroxidase enzyme was measured based on the increase in light absorbance due to the oxidation of guaiacol in the presence of hydrogen peroxide at a wavelength of 470 nm, using a JENWAY model 7315 spectrophotometer [49]. The measurements were taken over a period of 1 min, with 10 s intervals. The reaction mixture consisted of 50 µL of enzyme extract, 2.9 mL of 10 mM potassium phosphate buffer (pH 7), and 0.5 mL of 20 mM guaiacol. The reaction was initiated by adding 20 µL of 40 mM hydrogen peroxide. Using an extinction coefficient of 26.6 mM⁻¹cm⁻¹, the enzyme activity was calculated and reported as micromoles of oxidized guaiacol per minute per gram of fresh sample weight.

$$\mathrm{POD}\;\mathrm{Enzyme}\;\mathrm{Activity}\;({\mathrm{Mm}}^{-1}{\mathrm{cm}}^{-1}\mathrm{FW.min})=\frac{\mathrm{OD}(n)\;-\;\mathrm{OD}(0)}{\mathrm{Dilition}\;\mathrm{factor}\;\times \;\mathrm{FW}\;\times \;\mathrm{time}\;\times \;Ɛ}$$

where:

OD(n) and OD(0) represent the absorbance at time n and time zero, respectively,

FW is the fresh weight of the sample,

The dilution factor accounts for any sample dilution,

Time represents the reaction duration in minutes, and

Ɛ is the extinction coefficient of hydrogen peroxide at 240 nm.

### 4.5. Statistical Analyses

The statistical analysis of the collected data was performed using SPSS version 12. The means were compared using Duncan’s multiple range test at a 5% significance level, and graphs were created using Microsoft Excel.

## 5. Conclusions

The results of this study indicate that the application of the mycorrhizal fungus *Claroideoglomus etunicatum* significantly enhances the plant’s resistance to salt stress. Notably, IAA also has a positive impact on increasing the plant’s resistance to salt stress, and there is a synergistic effect between mycorrhiza and IAA in mitigating the negative impacts of salt stress. However, it should be noted that this study was conducted on a halophyte plant, and caution is needed when generalizing these findings to other plants. The scientific contribution of this article lies in explaining the mechanisms activated by IAA treatment and mycorrhizal fungi in the *Vetiver* plant, thereby enhancing its resistance to salinity. Additionally, the results of this research can serve as a foundation for advanced studies aimed at mitigating the effects of climate change and ensuring global food security.

## Figures and Tables

**Table 1:** Impact of salinity, IAA, and *C. etunicatum* on Na content of vetiver.

Treatment			Shoot Na Content (mg pot^−1^)	Root Na Content (mg pot^−1^)	Soil Na Concentration (mg pot^−1^)
Fungi	IAA	EC			
No inoculation fungi	Inoculation IAA	0	18.31 ± 0.25 gh	19.64 ± 5.45 ij	17.48 ± 0.41 l
		8	21.54 ± 7.37 efg	24.15 ± 20.81 hi	189.9 ± 0.41 i
		16	26.39 ± 18.66 d	28.41 ± 2.17 gh	571.9 ± 0.37 f
		24	32.12 ± 25.15 c	35.23 ± 58.62 f	853.8 ± 0.59 b
	No inoculation IAA	0	17.31 ± 0.33 h	17.52 ± 10.57 j	18.87 ± 0.44 k
		8	20.68 ± 33.10 fgh	18.90 ± 25.93 j	196.0 ± 0.30 h
		16	23.47 ± 8.31 def	24.97 ± 49.46 h	574.3 ± 1.02 e
		24	32.35 ± 48.50 c	28.93 ± 15.21 gh	861.4 ± 1.35 a
Inoculation fungi	Inoculation IAA	0	20.46 ± 0.47 fgh	40.74 ± 5.29 e	16.58 ± 0.18 l
		8	25.38 ± 43.80 d	40.03 ± 57.75 e	185.3 ± 0.24 j
		16	32.98 ± 21.68 c	51.44 ± 7.93 c	569.4 ± 0.45 d
		24	61.94 ± 2.95 a	68.03 ± 14.69 a	848.2 ± 1.49 c
	No inoculation IAA	0	19.81 ± 0.96 gh	33.14 ± 17.68 fg	17.47 ± 0.22 l
		8	24.11 ± 17.02 de	35.37 ± 2.64 f	190.1 ± 0.18 i
		16	33.01 ± 3.99 c	46.17 ± 2.50 d	572.9 ± 0.11 f
		24	54.74 ± 38.24 b	59.89 ± 7.57 b	851.9 ± 0.17 c

Note: each value represents the mean ± SD (*n* = 3). IN each column, different lowercase letters indicate significant differences among treatments (Duncan’s multiple range test; *p* < 0.05).

**Table 2:** Impact of salinity, IAA, and *C. etunicatum* on K uptake content of vetiver.

Treatment			Shoot K Uptake (mg pot^−1^)	Root K Uptake (mg pot^−1^)	Soil K Concentration (mg/kg)
Fungi	IAA	EC			
No inoculation fungi	Inoculation IAA	0	211.0 ± 0.26 c	48.19 ± 0.10 gh	5.26 ± 0.82 j
		8	210.0 ± 0.24 c	47.36 ± 0.26 gh	6.16 ± 0.65 hi
		16	121.5 ± 0.28 ef	40.05 ± 0.21 ij	6.50 ± 0.76 gh
		24	68.86 ± 0.13 h	32.13 ± 0.11 kl	8.73 ± 0.23 c
	No inoculation IAA	0	199.2 ± 0.32 c	43.08 ± 0.18 hi	4.60 ± 0.80 k
		8	200.6 ± 0.19 c	38.70 ± 0.30 ijk	5.66 ± 0.70 jk
		16	109.2 ± 0.24 g	35.40 ± 0.10 jk	6.23 ± 0.18 hi
		24	66.56 ± 0.33 h	27.64 ± 0.25 l	8.13 ± 0.40 d
Inoculation fungi	Inoculation IAA	0	238.9 ± 0.64 b	90.88 ± 0.07 a	5.86 ± 0.98 ij
		8	250.9 ± 0.49 a	76.38 ± 0.36 bc	7.10 ± 0.52 ef
		16	153.2 ± 0.04 d	72.20 ± 0.03 bc	7.76 ± 0.89 d
		24	130.1 ± 0.21 e	60.55 ± 0.10 ef	10.86 ± 0.37 a
	No inoculation IAA	0	229.8 ± 0.12 b	78.32 ± 0.11 b	5.86 ± 0.17 ij
		8	237.0 ± 0.18 b	69.63 ± 0.21 cd	6.70 ± 0.56 fg
		16	142.3 ± 0.18 d	64.91 ± 0.27 de	7.26 ± 0.39 e
		24	114.0 ± 0.30 fg	54.46 ± 0.07 fg	10.36 ± 0.54 b

Note: each value represents the mean ± SD (*n* = 3). IN each column, different lowercase letters indicate significant differences among treatments (Duncan’s multiple range test; *p* < 0.05).

**Table 3:** Impact of salinity, IAA, and *C. etunicatum* on P content of vetiver.

Treatment			Shoot P Uptake (mg pot^−1^)	Soil P Concentration (mg/kg)
Fungi	IAA	EC		
No inoculation fungi	Inoculation IAA	0	23.01 ± 0.12 c	20.42 ± 0.06 i
		8	21.27 ± 0.16 de	22.59 ± 0.11 g
		16	12.58 ± 0.18 g	24.51 ± 0.19 e
		24	5.85 ± 0.09 j	27.51 ±0.19 a
	No inoculation IAA	0	21.46 ± 0.11 d	18.71 ± 0.24 j
		8	20.26 ± 0.21 e	20.30 ± 0.25 i
		16	11.11 ± 0.16 h	22.51 ± 0.32 g
		24	5.63 ± 0.12 j	25.76 ± 0.08 c
Inoculation fungi	Inoculation IAA	0	26.04 ± 0.04 a	22.35 ± 0.11 g
		8	25.78 ± 0.12 a	23.06 ± 0.06 f
		16	15.87 ± 0.02 f	24.94 ± 0.05 d
		24	11.25 ± 0.22 h	27.61 ± 0.14 a
	No inoculation IAA	0	25.03 ± 0.22 ab	21.56 ± 0.09 h
		8	24.33 ± 0.08 b	21.70 ± 0.18 h
		16	14.75 ± 0.04 f	23.28 ± 0.14 f
		24	9.78 ± 0.11 i	26.56 ± 0.09 b

Note: each value represents the mean ± SD (*n* = 3). IN each column, different lowercase letters indicate significant differences among treatments (Duncan’s multiple range test; *p* < 0.05).

**Table 4:** Impact of salinity, IAA, and *C. etunicatum* on micro-elements content of vetiver shoot.

Treatment			Shoot Fe Concentration (mg/kg)	Shoot Zn Concentration (mg/kg))	Shoot Cu Concentration (mg/kg)	Shoot Mn Concentration (mg/kg)
Fungi	IAA	EC				
No inoculation fungi	Inoculation IAA	0	79.48 ± 0.33 b	43.19 ± 0.03 n	36.72 ± 0.08 b	55.96 ± 0.10 bc
		8	64.47 ± 0.40 f	55.43 ± 0.32 j	30.56 ± 0.12 f	54.00 ± 0.02 cd
		16	55.61 ± 0.17 j	69.52 ± 0.17 f	25.43 ± 0.06 j	37.90 ± 0.04 g
		24	42.49 ± 0.21 n	85.51 ± 0.32 b	18.53 ± 0.07 n	28.06 ± 0.06 j
	No inoculation IAA	0	76.43 ± 0.26 d	40.29 ± 0.17 p	36.72 ± 0.11 d	54.23 ± 0.11 d
		8	61.89 ± 0.32 h	52.32 ± 0.09 l	29.46 ± 0.52 h	53.46 ± 0.03 d
		16	52.26 ± 0.23 l	66.68 ± 0.25 h	24.76 ± 0.08 l	36.83 ± 0.05 g
		24	39.51 ± 0.32 p	82.13 ± 0.10 d	17.75 ± 0.06 p	27.33 ± 0.07 j
Inoculation fungi	Inoculation IAA	0	80.51 ± 0.24 a	45.20 ± 0.09 m	37.30 ± 0.08 a	58.80 ± 0.05 a
		8	65.41 ± 0.27 e	56.28 ± 0.16 i	31.24 ± 0.15 e	55.13 ± 0.03 cd
		16	56.46 ± 0.29 i	70.52 ± 0.13 e	26.15 ± 0.05 i	44.06 ± 0.03 e
		24	43.38 ± 0.35 m	86.56 ± 0.15 a	19.11 ±0.15 m	33.16 ± 0.13 h
	No inoculation IAA	0	77.98 ± 0.15 c	41.90 ± 0.04 o	36.38 ± 0.10 c	57.40 ± 0.11 ab
		8	63.31 ± 0.18 g	53.18 ± 0.05 k	30.22 ± 0.05 g	54.66 ± 0.05 cd
		16	53.15 ± 0.04 k	67.44 ± 0.37 g	25.13 ± 0.11 k	41.13 ± 0.02 f
		24	40.26 ± 0.10 o	83.52 ± 0.12 c	18.27 ± 0.08 o	31.26 ± 0.05 i

Note: each value represents the mean ± SD (*n* = 3). IN each column, different lowercase letters indicate significant differences among treatments (Duncan’s multiple range test; *p* < 0.05).

**Table 5:** Impact of salinity, IAA, and *C. etunicatum* on micro-elements content of vetiver root.

Treatment			Root Zn Concentration (mg/kg))	Root Cu Concentration (mg/kg)	Root Mn Concentration (mg/kg)
Fungi	IAA	EC			
No inoculation fungi	Inoculation IAA	0	27.89 ± 0.08 b	36.93 ± 0.45 ab	46.19 ± 0.06 b
		8	22.08 ± 0.03 f	28.18 ± 0.45 c	37.83 ± 0.04 f
		16	15.82 ± 0.21 j	21.37 ± 0.02 de	29.87 ± 0.03 j
		24	9.92 ± 0.03 m	14.89 ± 0.03 gh	19.32 ± 0.07 n
	No inoculation IAA	0	27.40 ± 0.04 d	36.16 ± 0.06 b	45.71 ± 0.25 d
		8	21.35 ± 0.03 h	27.44 ± 0.05 c	37.34 ± 0.04 h
		16	15.43 ± 0.02 k	20.46 ± 0.02 f	27.28 ± 0.02 l
		24	9.57 ± 0.01 o	14.19 ± 0.06 h	18.85 ± 0.05 p
Inoculation fungi	Inoculation IAA	0	28.17 ± 0.04 a	37.20 ± 0.03 a	46.44 ± 0.05 a
		8	22.39 ± 0.04 e	27.56 ± 1.74 c	38.12 ± 0.03 e
		16	16.21 ± 0.04 i	21.90 ± 0.06 d	30.29 ± 0.03 i
		24	10.19 ± 0.02 l	15.43 ± 0.03 g	19.73 ± 0.07 m
	No inoculation IAA	0	27.64 ± 0.04 c	36.66 ± 0.02 ab	45.93 ± 0.04 c
		8	21.89 ± 0.02 g	27.89 ± 0.04 c	37.68 ± 0.03 g
		16	15.71 ± 0.02 j	20.93 ± 0.03 ef	29.52 ± 0.07 k
		24	9.73 ± 0.02 n	14.60 ±0.04 h	19.08 ± 0.03 o

Note: each value represents the mean ± SD (*n* = 3). IN each column, different lowercase letters indicate significant differences among treatments (Duncan’s multiple range test; *p* < 0.05).

**Table 6:** Effect of NaCl, IAA, and *C. etunicatum* on the antioxidative enzymes of vetiver.

Treatment			Plant Proline (Micromol per Gram Fresh Weight of Plant Leaves)	Catalase Enzyme Activity (Units per Milligram of Plant Fresh Weight)	Superoxide Dismutase Enzyme Activity (Units per Milligram of Fresh Plant Leaf Weight)	Peroxidase Enzyme Activity (Units per Milligram of Plant Fresh Weight)
Fungi	IAA	EC				
No inoculation fungi	Inoculation IAA	0	9.793 ± 0.36 gh	41.83 ± 1.38 h	62.85 ± 0.40 c–f	13.59 ± 0.19 k
		8	9.633 ± 1.09 gh	46.72 ± 1.61 ef	59.00 ± 35.08 ef	15.31 ± 0.18 i
		16	16.34 ± 0.39 cde	50.26 ± 0.25 d	91.34 ± 1.42 abc	18.17 ± 0.24 d
		24	19.20 ± 0.72 b	57.60 ± 0.52 b	107.10 ± 0.28 ab	19.38 ± 0.35 b
	No inoculation IAA	0	11.25 ± 0.09 f	45.30 ± 0.17 fg	65.41 ± 0.28 cde	14.42 ± 0.16 j
		8	10.00 ± 0.43 g	49.20 ± 0.88 d	80.82 ± 0.56 b–e	16.54 ± 0.23 g
		16	16.74 ± 0.12 cd	52.70 ± 0.26 c	94.33 ± 1.15 ab	18.46 ± 0.29 cd
		24	20.73 ± 1.10 a	60.26 ± 0.20 a	111.1 ± 0.85 a	20.34 ± 0.13 a
Inoculation fungi	Inoculation IAA	0	9.16 ± 0.28 gh	34.80 ± 0.52 j	61.39 ± 0.40 def	13.10 ± 0.16 l
		8	8.83 ± 0.20 h	42.03 ± 1.91 h	35.70 ± 35.00 f	14.47 ± 0.23 j
		16	15.38 ± 0.12 e	43.13 ± 0.65 gh	88.18 ± 0.17 a–d	16.96 ± 0.19 f
		24	16.96 ± 0.20 cd	48.53 ± 1.42 de	104.80 ± 0.28 ab	18.65 ± 0.25 c
	No inoculation IAA	0	9.45 ± 0.79 gh	37.23 ± 1.25 i	63.47 ± 0.10 c–f	13.50 ± 0.10 k
		8	9.16 ± 0.85 gh	44.00 ± 1.00 gh	58.66 ± 34.61 ef	15.72 ± 0.13 h
		16	16.02 ± 0.18 de	44.90 ± 0.36 fg	91.47 ± 0.50 abc	17.48 ± 0.21 e
		24	17.23 ± 0.49 c	53.96 ± 3.32 c	106.20 ± 0.25 ab	19.40 ± 0.09 b

Note: each value represents the mean ± SD (*n* = 3). IN each column, different lowercase letters indicate significant differences among treatments (Duncan’s multiple range test; *p* < 0.05).

**Table 7:** Physicochemical characteristics of the soil used in the present experiment.

Feature	The Amount
Sand	57.72%
Silt	12.56%
Clay	29.72%
Texture class	Sandy clay loam
pH saturated dough	7.6
Electrical conductivity of the saturated extract	2.15 dS/m
Cation exchange capacity	18 cmol^+^/kg
Organic matter	1.3%
Total N	0.07%
P extractable by NaHCO_3_	15 mg/kg
K extractable by C_2_H_7_NO_2_	420 mg/kg
Extractable Cu with DTPA	5.1 mg/kg
Extractable Mn with DTPA	0.6 mg/kg
Extractable Zn with DTPA	3 mg/kg
Extractable Cd with DTPA	1.3 mg/kg
Microbial respiration	5.2 mg CO_2_-C.Kg^−1^.h^−1^
Soil microbial biomass carbon	15.15 mg of C/kg of soil

## Data Availability

Data is contained within the article.

## Appendix A

**Table A1:** Analysis of variance for effect of individual treatments and interaction on the morphological and physiological characteristics of vetiver grass. Salt stress (S), fungi and IAA (M), Interaction (S × M). Plant height (PH), Fresh weight (FW), Root/Shoot (R/Sh), Dry weight (DW), Root dry weight (RDW), Electrical conductivity (EC), Shoot sodium (ShNa), Root sodium (RNa), Soil sodium (SNa), Shoot potassium (ShK), Root potassium (RK), Soil potassium (SK), Shoot phosphorous (ShP), Soil phosphorous (SP), Shoot Iron (ShFe), Shoot Zink (ShZn), Shoot copper (ShCu), Shoot manganese (ShMn), Root Zink (RZn), Root copper (RCu), Root manganese (RMn), Proline (Pr), Catalase (CAT), Dismutase (DIS), Peroxidase (POD). (** represents significance at *p* < 0.01; ns means non-significant effect).

	S	M	S × M
PH (cm)	ns	**	ns
FW (g)	**	**	**
R/Sh	**	**	**
DW (g)	**	**	**
RDW (g)	ns	**	ns
EC (ds/m)	**	ns	ns
ShNa (mg pot^−1^)	**	**	**
RNa (mg pot^−1^)	**	**	**
SNa (mg/kg)	**	**	**
ShK (mg pot^−1^)	**	**	**
RK (mg pot^−1^)	**	**	ns
SK (mg/kg)	**	**	**
ShP (mg pot^−1^)	**	**	ns
SP (mg/kg)	**	**	**
ShFe (mg/kg)	**	**	ns
ShZn (mg/kg)	**	**	**
ShCu (mg/kg)	**	**	ns
ShMn (mg/kg)	**	**	ns
RZn (mg/kg)	**	**	**
RCu (mg/kg)	**	**	**
RMn (mg/kg)	**	**	**
Pr (µmol/g)	**	**	**
CAT (U/mg)	**	**	**
DIS (U/mg)	**	ns	ns
POD (U/mg)	**	**	**
